# Laparoscopic antireflux surgery or PPIs in the management of reflux-related esophageal stricture

**DOI:** 10.1007/s00464-022-09564-5

**Published:** 2022-09-15

**Authors:** Zhi-tong Li, Xiang-lin Kong, Rui Zhang, Jian-ning Yao, Chun-xia Li, Xin-wei Han, Zhong-gao Wang, Kang-dong Liu, Feng Ji

**Affiliations:** 1 Department of Gastroenterology, The First Affiliated Hospital, Zhengzhou University, No. 1, East Jian She Road, Zhengzhou, 450052 Henan Province People’s Republic of China; 2 Shandong University of Traditional Chinese Medicine, No. 4655, University Road, Jinan, 250355 Shandong Province People’s Republic of China; 3 Department of Respiratory and Critical Medicine, The First Affiliated Hospital, Zhengzhou University, No. 1, East Jian She Road, Zhengzhou, 450052 Henan Province People’s Republic of China; 4 Department of Interventional Radiology, The First Affiliated Hospital, Zhengzhou University, No. 1, East Jian She Road, Zhengzhou, 450052 Henan Province People’s Republic of China; 5 School of Basic Medical Science, Zhengzhou University, Zhengzhou, 450001 Henan Province People’s Republic of China

**Keywords:** Gastroesophageal reflux disease, Esophageal stricture, Balloon catheter dilation, Antireflux surgery

## Abstract

**Background:**

Gastroesophageal reflux disease (GERD) is often associated with esophageal stricture, particularly benign esophageal stricture. We aimed to evaluate the effects of balloon catheter dilation (BD) combined with laparoscopic fundoplication (LF) surgery and proton pump inhibitors (PPIs) in patients with reflux-induced esophageal strictures.

**Methods:**

We retrospectively analyzed 116 patients with reflux-induced benign esophageal strictures who underwent balloon dilatation therapy combined with PPIs (BD-PPIs group, *n* = 58) and balloon dilatation combined with LF (BD-LF group, *n* = 58). Patients were followed up for 24 months. The outcomes of the patients were monitored, including clinical success, symptom improvement, adverse events, and the frequency of esophagitis.

**Results:**

At the latest follow-up, the rate of clinical success was higher in BD-LF group than in BD-PPIs group (80.4% vs. 57.7%, *P* = 0.011). The patients in the BD-PPIs group required more dilation sessions to achieve successful dilation, as compared to those in the BD-LF group (2.1 ± 1.2 vs. 0.7 ± 0.8, *P* < 0.001). The DeMeester score, number of reflux episodes for which pH was < 4, and lower esophageal sphincter pressure were significantly better in the BD-LF group than in the BD-PPIs group (all *P* < 0.001). The incidence of reflux esophagitis was higher in the BD-PPIs group than in the BD-LF group, at 24 months (58.8% vs. 18.2%, *P* = 0.003).

**Conclusions:**

Balloon dilatation with concomitant LF is effective and safe for esophageal stricture secondary to GERD. Moreover, antireflux surgery techniques, such as Nissen or Toupet procedure, should be added for reflux-induced benign esophageal stricture.

Benign esophageal strictures are commonly observed in people, and the causes are numerous and complex. Reflux strictures are still common in western societies (rarely resistant nowadays) though perhaps less so in East Asia. Gastroesophageal reflux disease (GERD) is a common medical condition, but it can cause a variety of serious complications, including esophageal mucosal inflammation, ulcers, strictures, and even esophageal cancer [1, 2]. The reflux of gastric acid into the esophagus leads to changes in the esophageal mucosa, which produces clinical symptoms and various complications [3]. Patients with esophagitis reportedly have 8 times higher risk of developing strictures [4]. Due to lack of sufficient attention, many patients with gastroesophageal reflux gradually develop esophageal strictures.

The treatment options for esophageal strictures include mechanical dilatation with a bougie or balloon, stent placement, and autologous keratinocyte implantation [5]. Balloon catheter dilation (BD) is a common non-surgical treatment for benign esophageal strictures. Most benign esophageal strictures can be treated successfully via dilatation. However, approximately 30% of esophageal benign strictures exhibit a very high propensity to recur after any kind of mechanical treatment [6]. Esophageal strictures secondary to reflux esophagitis may be a cause of the failure of dilation therapy. Fundoplication is an important part of laparoscopic antireflux surgery (LARS) that is often performed for medication-refractory GERD [7]. To avoid such undesirable outcomes, the awareness of GERD should be improved.

To our knowledge, no studies have analyzed the potential preventative benefits of LARS therapy, or assessed whether it is long-term safe and effective for resistant or recurrent esophageal strictures after dilatation therapy. In the present study, we examined the effects of dilatation therapy combined with proton pump inhibitors (PPIs) and simultaneous therapy with both BD and laparoscopic fundoplication (LF, Nissen, or Toupet) for esophageal strictures caused by GERD.

## Materials and methods

### Ethical statement

Written informed consent was obtained from all subjects. The ethics committee of the First Affiliated Hospital of Zhengzhou University approved this retrospective cohort study.

### Study population

We retrospectively reviewed and analyzed the patients who exhibited symptoms of stricture from January 2014 to December 2018. Patients were eligible for enrollment in this study if they had a previous history of gastroesophageal reflux, such as heartburn, regurgitation, substernal pain, suprasternal discomfort, and so on. As time progressed, dysphagia verging on episodic aphagia became more frequent and culminated in persistent dysphagia along with varying degrees of weight loss. On previous endoscopic examinations, they indicated reflux esophagitis (grade A to D, according to the Los Angeles classification [8]), and benign esophageal strictures were found on current examinations.

Patients were excluded if they had malignancy, anastomotic stricture, chemical corrosive esophageal injury, eosinophilic esophagitis, or major motility disorders by the Chicago Classification [9] (such as esophageal spasm, absent peristalsis, hypercontractile esophagus, achalasia, esophagogastric junction outflow obstruction). Patients who exhibited symptoms of stricture, but did not show any upper gastrointestinal stricture on endoscopic examination were also excluded.

The esophageal stricture was defined as an esophageal diameter < 11 mm, rather than the inability to pass the gastroscope (with a diameter of 9.8–11.0 mm) or the inability to achieve or maintain a diameter of 14 mm despite dilatation every 2–4 weeks [10, 11]. According to the length of the esophageal stricture and the condition of the lumen, esophageal stricture was divided into short esophageal stricture (≤ 2 cm) and long esophageal stricture (> 2 cm) [12].

**Fig. 1:**
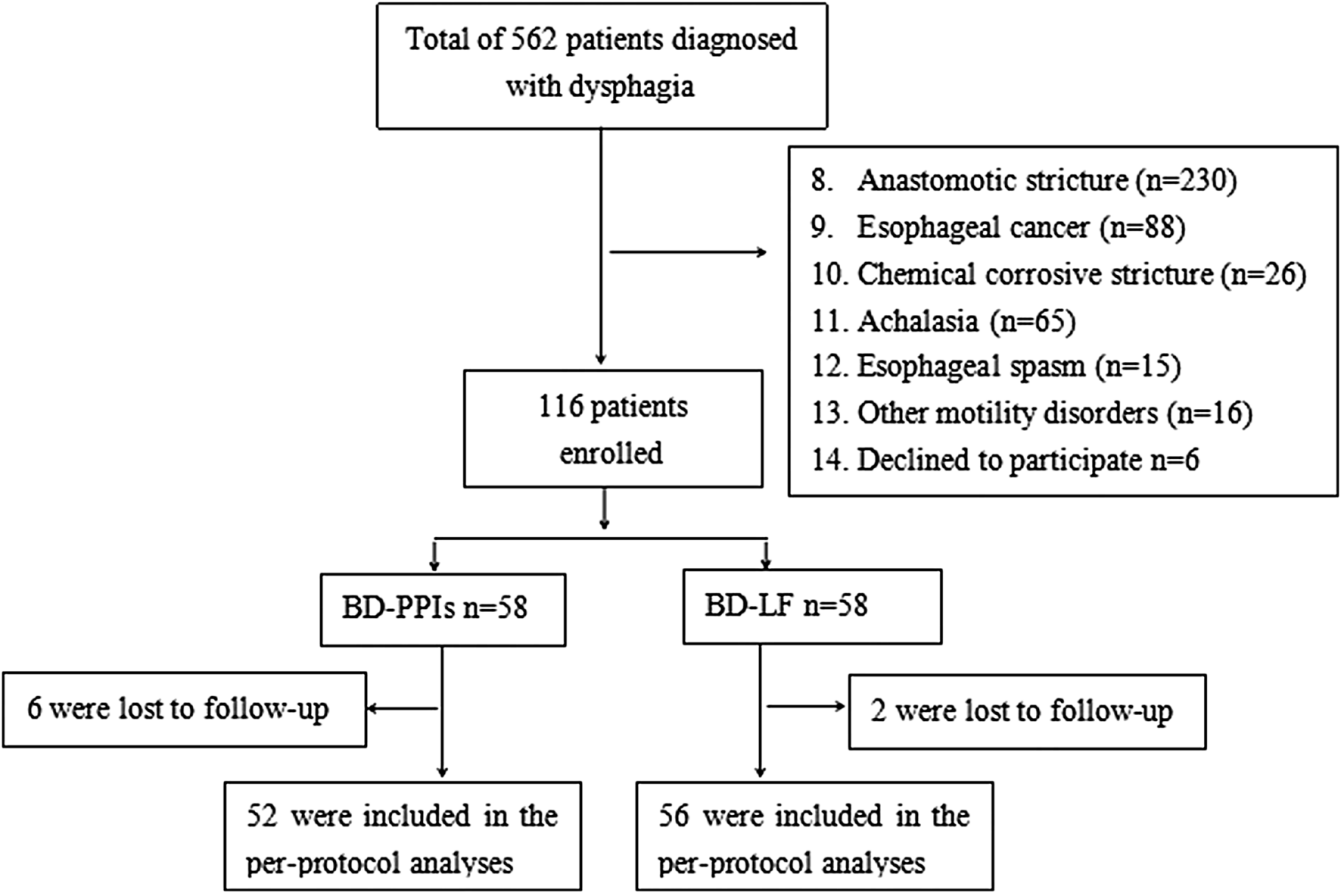
Flow chart of patients enrolled in this study. *BD* balloon dilation; *PPIs* proton pump inhibitors; *LF* laparoscopic fundoplication

According to the patient's individual needs (e.g. cost, cosmetic effect, and complication), the patients were divided into two treatment groups: BD-PPIs group and BD-LF group (Fig. 1).

### Surgical technique

We performed dilation with radiographic guidance using a controlled radial expansion balloon catheter (diameter 10 mm-36 mm, Microvasive, Boston Scientific Corporation, USA).

**Fig. 2:**
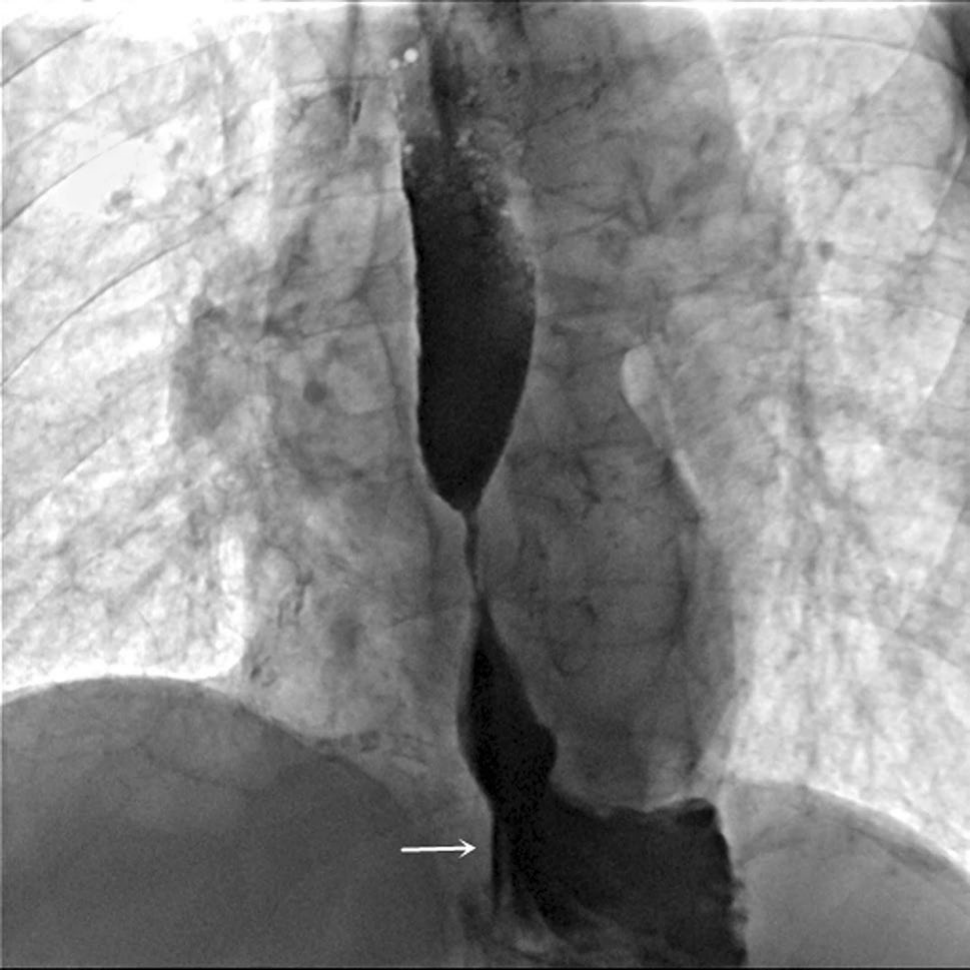
Contrast swallow showed a severe stricture and a small hiatus hernia distal to the narrow contrast column (white arrow)

The patient fasted for 12 h. The oral contrast agent was then passed through the esophageal under radiography to determine the length and shape of the esophageal stricture (Fig. 2). Thereafter, an appropriate balloon catheter diameter (10-36 mm) was selected and the catheter was introduced via the oral route to the proximal limit of the stricture under the guidance of the guidewire. A graded balloon was then passed through the stricture. The balloon was inflated by injecting a contrast agent to the recommended pressure for 60 s. The elimination of “lumbar type” stricture was an indicator of esophageal balloon dilatation.

The patients were kept in a fasting state for 4 h after the procedure, and PPIs (double dosage of omeprazole or esomeprazole) were prescribed to suppress gastric acid production. Double use of PPIs is the recommended method for severe GERD [13]. Patients with severe esophageal stricture underwent 2 sessions per week. The initial balloon diameter was chosen based on the stricture diameter. Serial dilations were performed by gradually increasing the balloon diameters, until solid or semisolid food could be tolerated.

If active ulceration was noted within the narrow segment under the endoscopy, the dilation procedure could be postponed. PPIs and sucralfate were prescribed, and the patient was re-evaluated after 4 weeks. After one month of success with dilation, a control group of patients was treated with LF or LF combined with hiatal hernia repair (LF-HHR), as described in our previous studies [14]. The surgical technique of laparoscopic surgical procedure was performed by dissecting a 3–4 cm of esophageal reposition and suturing esophageal hiatus intermittently with non-absorbable lines. If 360-degree Nissen fundoplication was added, a full-circle valve was formed from the posterior and anterior aspects of the fundus and sutured together using three separate nonabsorbable 2–0 sutures. If 270-degree Toupet fundoplication was added, a three-quarter-circle valve was formed.

The time node we assigned group was 1 month after successful balloon expansion. For those patients who did not respond to dilation, serial dilations were performed by gradually increasing the balloon diameters, until solid or semisolid food could be tolerated. After one month of success with dilation, the patients were assigned to two treatment groups. In BD-LF group, PPIs were no longer applied after discharge. In BD-PPIs group, a double dosage of PPIs continued to be prescribed.

### Evaluation of the outcome

The primary outcomes were comparisons of clinical success, symptoms, and adverse events during postoperative follow-up. Patient-reported outcomes were accomplished using outpatient visits or telephone.

The starting point for follow-up was the non-recurrence of esophageal stricture within 1 month after BD. The primary endpoint was an improvement of Eckardt’s symptom score during the 2-year follow-up, which clinical success was defined as Eckardt’s symptom score of 3 or less without the use of additional treatments. A 4-point scale ranging (0–3) is applied to assess the level of dysphagia, regurgitation, chest pain, and weight loss according to the Eckardt symptom score [15]. The maximum Eckardt symptom score is 12 points, with higher scores indicating more severe symptoms. The number of repeat dilations was also evaluated for patients with symptom relapse and proven stricture recurrence during the 2-year follow-up.

Secondary study endpoints included the grading of endoscopic reflux lesions, assessment of esophageal function using high-resolution esophageal manometry (HRM), and 24-h PH monitoring (at least 1 week after the discontinuation of PPIs), which were planned prior to treatment and after LARS for 3 and 24 months. Esophagogastroduodenoscopy (EGD) was performed to assess the degree of stricture and the extent of damage in the gastrointestinal tract. The DeMeester score (DMS), number of reflux episodes for which pH was < 4, and lower esophageal sphincter pressure (LESP) were evaluated by the 24-h PH monitoring and HRM. Contrast esophagography was also performed in patients who complained of dysphagia, or at 4 weeks after the last EGD in patients who were asymptomatic, in order to quantitatively assess the degree of stricture recovery.

Additional secondary endpoints included adverse events, such as bleeding, perforation or mucosal damage, prolonged pain, ulcerations that occurred during or after the procedure.

### Statistical analysis

Data analysis was performed using SPSS version 13.0 software (SPSS Inc, Chicago, IL, USA) and GraphPad InStat, version 3.06 (GraphPad Software, San Diego, CA, USA). Continuous variables were expressed as median or means ± standard deviations. Comparisons were made between the BD-PPIs group and BD-LF group, and between the preoperative and postoperative status using the chi-squared test, the Wilcoxon paired-samples test, or the *t-test*, as appropriate. Kaplan–Meier’s survival curves were plotted to display the endpoint of stricture recurrence. All statistical tests were considered significant when two-tailed *P* values were < 0.05.

## Results

### Demographic findings and clinical symptoms

Of 562 patients with dysphagia for this study, a total of 116 patients met the inclusion criteria (Fig. 1). Among these patients, 59.5% (*n* = 69) were men and 40.5% (*n* = 47) were women. The mean age was 56.8 ± 9.8 years (range, 40–80 years). The patients’ demographic and clinical characteristics in the two groups were listed in Table 1 and showed comparability between groups. All patients had a previous history of gastroesophageal reflux and reflux esophagitis Los Angeles grade A-D, with a mean duration of 9.2 ± 4.5 years (range, 2–25 years). 22 patients previously had one or two dilatations, then the strictures recurred again. Histologically, the infiltration of inflammatory cells—mainly neutrophils, lymphocytes, and plasma cells—and fibrosis were observed. Although erosion and ulceration were noted, no malignant findings were observed. Contrast esophagography showed the mean distance from the esophageal stricture to the incisor was 33.9 ± 3.5 cm, and 75.9% (88/116) cases were short-segment esophageal stricture; the mean length was 32.4 ± 8.4 mm (Table 1). In addition, 41.4% (48/116) of the patients showed hiatal hernia.

**Table 1:** Demographic and clinical characteristics of the study patients (*n* = 116)

Characteristics	BD-PPIs (*n* = 58)	BD-LF (*n* = 58)	*P*-value
Age (years) mean ± SD	56.6 ± 11.1	58.2 ± 10.1	0.459
Male, *n* (%)	38 (65.5)	31 (53.4)	0.186
BMI (kg/m^2^) mean ± SD	19.2 ± 2.6	19.6 ± 2.4	0.643
Duration of heartburn (years) mean ± SD	8.6 ± 3.8	10.3 ± 5.1	0.194
Previous dilatation, *n*	10	12	0.636
Eckardt symptom scores	7.3 ± 1.5	7.4 ± 1.5	0.751
Stricture distance from the incisors (cm)	33.8 ± 3.9	34.0 ± 3.2	0.847
Stricture length (mm)	32.1 ± 8.8	32.7 ± 8.2	0.823
Stricture diameter (mm)	3.7 ± 1.9	3.8 ± 1.9	0.914
Hiatal hernia, *n*	20	28	0.132

*BD* balloon dilation; *PPIs* proton pump inhibitors; *LF* laparoscopic fundoplication; *SD* standard deviation; *BMI* body mass index

### Intra- and postoperative outcomes

BD and LF or LF-HHR were successfully completed in all the patients, and the complications of BD included chest pain (*n* = 30; 25.9%), bleeding (*n* = 5; 4.3%), and esophageal perforation (*n* = 2; 1.7%). With fasting and supportive treatment via jejunal feeding for 1 month, the esophageal perforation healed in the cases. After LF or LF-HHR, 12 cases experienced complications, such as dysphagia, abdominal pain, or abdominal distension, but these issues fully or partially disappeared within 3–4 weeks. In addition, there was no difference with respect to dysphagia and hernia recurrence between LNF and LTF over time (not shown). All the patients survived the surgery during follow-up.

### Primary endpoint

**Fig. 3:**
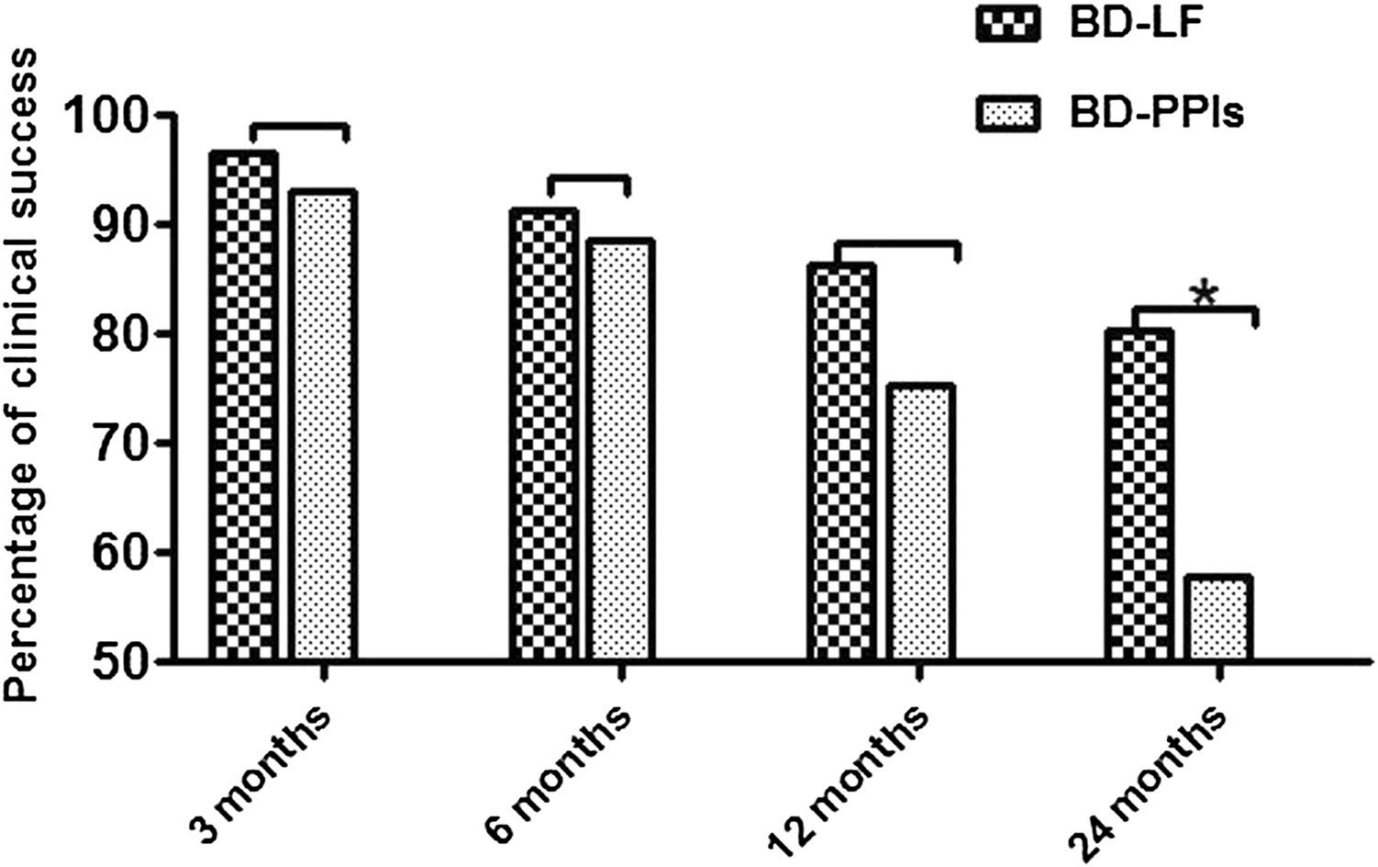
Rates of clinical success during the 2-year follow-up period. *BD* balloon dilation; *PPIs* proton pump inhibitors; *LF* laparoscopic fundoplication. The rate of clinical success was higher in BD-LF group than in BD-PPIs group. *P < 0.05

Over the 2-year follow-up period, a total of 108 patients satisfying the inclusion criteria were included in the final analysis. 8 patients were lost to follow-up (6 in BD-PPIs group, 2 in BD-LF group; Fig. 1). At 3 months after surgery, 54 of 58 patients (93.1%) in the BD-PPIs group and 56 of 58 patients (96.6%) in the BD-LF group had clinical success. The percentages of patients who had clinical success over time were shown in Fig. 3. By the end of the 2-year follow-up, the patients who had clinical success after the assigned intervention were 57.7% (30/52) in the BD-PPIs group and 80.4% (45/56) in the BD-LF group (*P* = 0.011; Fig. 3). The Eckardt symptom scores were lower after surgery as compared to the corresponding values before surgery (BD-PPIs group: 7.3 ± 1.5 vs. 3.9 ± 2.1, *P* < 0.001; BD-LF group: 7.4 ± 1.5 vs. 2.5 ± 1.6, *P* < 0.001; Tables 1 and 3). However, the Eckardt symptom scores were significantly higher in the BD-PPIs group than in the BD-LF group (*P* = 0.003; Table 3).

**Fig. 4:**
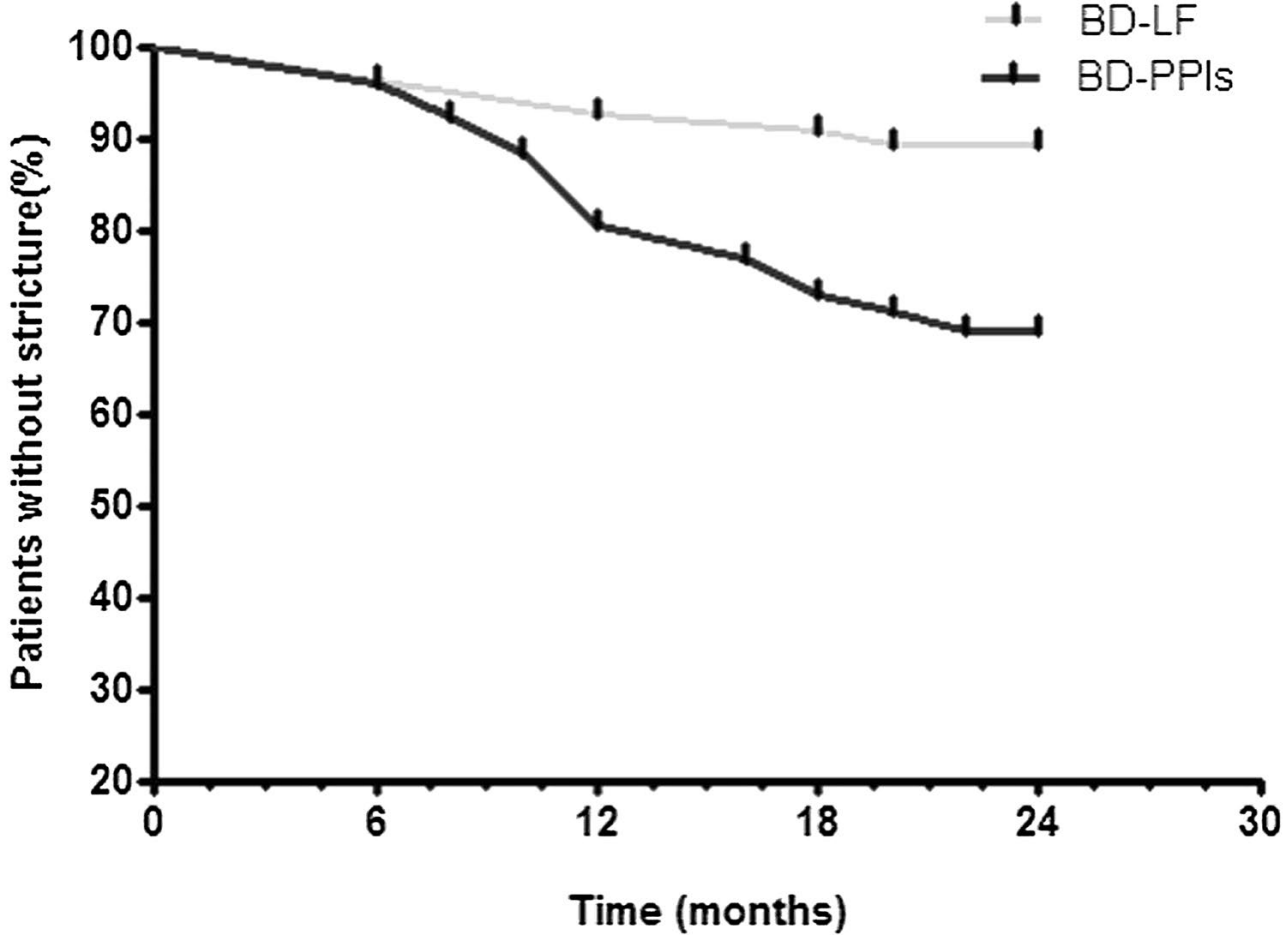
Kaplan–Meier survival curves showed stricture recurrence in the two study groups. *BD* balloon dilation; *PPIs* proton pump inhibitors; *LF* laparoscopic fundoplication

Kaplan–Meier’s curves of two groups for survival without stricture recurrence showed in Fig. 4. The average time of stricture recurrence was 14.0 ± 6.3 (6–20) months and 12.7 ± 4.8 (6–22) months in BD-LF group and BD-PPIs group (Fig. 4), respectively. Moreover, the patients in the BD-PPIs group required a greater number of dilation sessions to achieve successful dilation, as compared to those in the BD-LF group (2.1 ± 1.2 vs. 0.7 ± 0.8, *P* < 0.001; Table 3).

### Secondary endpoints

At 3 months after surgery, 77.6% (45/58) of BD-PPIs patients and 81.0% (47/58) of BD-LF patients could undergo the HRM and 24-h PH monitoring test. A significant improvement in the DMS, the number of reflux episodes with pH < 4, and LESP were noted in the BD-LF group. The mean DMS and number of reflux episodes with pH < 4 decreased from 50.4 ± 27.5 to 8.2 ± 4.1 and from 63.4 ± 31.5 to 20.6 ± 7.7 (all *P* < 0.001; Table 2) in the BD-LF group, respectively. The LESP also significantly increased from 2.8 ± 3.9 mmHg to 24.1 ± 7.4 mmHg (*P* < 0.001; Table 2) in the BD-LF group. By the end of the 2-year follow-up, these parameters did not change significantly. However, in the BD-PPIs group, the mean DMS and the number of reflux episodes with pH < 4 improved three months after surgery compared with preoperative (all *P *< 0.05; Table 2). With the follow-up time, there were no significant changes in these parameters compared with preoperative (all *P* > 0.05; Table 2). The LESP remained unchanged before and after follow-up. The DMS, number of reflux episodes with pH < 4, and LESP were significantly better in the BD-LF group than in the BD-PPIs group (all *P* < 0.001; Table 2).

**Table 2:** Objective evaluation of the study patients

Characteristics	Preoperative		3 months		24 months	
	BD-PPIs (*n* = 58)	BD-LF (*n* = 58)	BD-PPIs (*n* = 45)	BD-LF (*n* = 47)	BD-PPIs (*n* = 20)	BD-LF (*n* = 33)
DMS	48.3 ± 26.4	50.4 ± 27.5	31.2 ± 22.3	8.2 ± 4.1	38.4 ± 20.8	12.3 ± 4.6
Number of reflux episodes	69.2 ± 34.8	63.4 ± 31.5	52.0 ± 17.7	20.6 ± 7.7	58.7 ± 19.6	23.5 ± 6.8
LESP, mmHg	3.8 ± 5.7	2.8 ± 3.9	4.3 ± 4.8	24.1 ± 7.4	4.6 ± 4.2	22.3 ± 6.5
Esophagitis, no./total no. (%)	58	58	40/56(71.4)	32/54(59.3)	20/34(58.8)	4/22(18.2)
LA, A	0	0	4/56(7.1)	14/54(25.9)	6/34(17.6)	3/22(13.6)
LA, B	0	0	5/56(8.9)	10/54(18.5)	8/34(23.5)	1/22(4.5)
LA, C	41/58(70.7)	45/58(77.6)	23/56(41.1)	7/54(13.0)	6/34(17.6)	0/22(0)
LA, D	17/58(29.3)	13/58(22.4)	8/56(14.3)	1/54(1.9)	0/34(0)	0/22(0)

*BD* balloon dilation; *PPIs* proton pump inhibitors; *LF* laparoscopic fundoplication; *SD* standard deviation; *DMS* DeMeester score; *LESP* lower esophageal sphincter pressure (normal range:13–43 mmHg); *LA* Los Angeles classification

Among 110 and 56 patients in the intention-to-treat population who underwent EGD at 3 months and 2 years, respectively, the incidence of reflux esophagitis (all grades) was higher in the BD-PPIs group than in the BD-LF group, both at 3 months (71.4% vs. 59.3%; *P* = 0.180) and 24 months (58.8% vs. 18.2%, *P* = 0.003; Table 2). As summarized descriptively, high-grade esophagitis (Los Angeles Classification grade C and D) was observed at 3 months in 31 of 56 patients (55.4%) in the BD-PPIs group and 8 of 54 patients (14.8%) in the BD-LF group and 24 months in 6 of 34 patients (17.6%) in the BD-PPIs group and 0 patients in the BD-LF group (all *P* < 0.05; Table 2). The mean minimum diameter of the stenotic lumens just after therapy was greater in BD-LF group than in BD-PPIs group (19.5 ± 4.9 mm vs. 14.7 ± 2.0 mm, respectively, *P *= 0.001; Table 3).

**Table 3:** Outcomes at the end of the follow-up period

Characteristics	BD-PPIs (*n* = 52)	BD-LF (*n* = 56)	*P*-value
Stricture diameter (mm)	14.7 ± 2.0	19.5 ± 4.9	0.001
Eckardt symptom scores	3.9 ± 2.1	2.5 ± 1.6	0.003
Number of dilation sessions	2.1 ± 1.2	0.7 ± 0.8	< 0.001

*BD* balloon dilation; *PPIs* proton pump inhibitors; *LF* laparoscopic fundoplication

## Discussion

This study was the first to evaluate the effect of routine fundoplication or combined with HHR as an adjunct to BD in patients with reflux-related benign esophageal strictures. We assessed clinical success, symptom improvement, adverse events, and the frequency of esophagitis during postoperative follow-up.

Most esophageal strictures are malignant, and benign esophageal strictures are rarely noted [16]. A meta-analysis showed peptic strictures accounted for 25.0% of benign esophageal strictures, corrosive strictures occupied 12.5% [17]. Peptic esophageal strictures due to chronic reflux-associated inflammation were found in 0.2% of examined patients [18]. Prior dysphagia, GERD, hiatal hernia, peptic ulcer disease, and heavy alcohol use were associated with an increased risk of stricture [16].

In this study, all the patients experienced symptoms of gastroesophageal reflux for several years. Dysphagia verging on episodic aphagia became more frequent and culminated in persistent dysphagia over a relatively short period in the present study. Hiatal hernia also was noted in 41.4% of patients. Erosion and ulceration were observed, but no malignant findings were noted. Although the HRM and 24-h pH monitoring were required for the diagnosis of GERD, these tests were not performed because the catheters would not be able to pass through the esophagus due to the severe strictures. So, after satisfactory dilatation for 1 week and termination of anti-acid medication for 1 week, these tests were performed. Ineffective esophageal motility was screened by esophageal manometry, which was believed to be an important pathologic feature of both GERD [19] and dysphagia [20]. The DMS, number of reflux episodes with pH < 4, and LESP were significantly abnormal (Table 2). These patients met the criteria for the diagnosis of benign esophageal strictures secondary to GERD.

With regard to the mechanism of stricture formation from reflux esophagitis, the lower esophageal sphincter, or valvular mechanism, in these cases were believed to be destroyed and incapable of preventing gastric reflux. Gastric acid reflux leads to inflammation in the lamina propria, which is then disrupted and results in stricture formation [21].

Severe GERD is frequently observed in cases of severe esophageal stricture due to corrosive esophagitis or long-gap esophageal atresia. Cicatricial shortening of the esophagus is an important cause of GERD and consequent stricture worsening. Higuchi et al. [22] reported that 91.4% of reflux-induced esophageal ulcers were located in the lower esophagus. Moreover, most of the esophageal strictures caused by reflux esophagitis develop at the lower end of the esophagus [23] and were short segments [24]. Our results showed that reflux-induced strictures were located in the lower esophagus, with a mean distance of 33.9 ± 3.5 cm from the incisors. Barium contrast esophagography showed that 75.9% (88/116) of the esophageal strictures were short segments, and the mean length was 32.4 ± 8.4 mm.

The treatment options for esophageal stricture include mechanical dilatation with a bougie or balloon, stent placement, and autologous keratinocyte implantation [5]. The indications for each treatment depend on the operator’s experience and preferences, as well as the etiology of the esophageal stricture. Many studies have reported the successful use of balloon dilation to treat esophageal stricture [25, 26]; surgical resection is rarely performed due to the associated high morbidity and mortality rates [27]. However, recurrent strictures occur within 1 year in one-third of cases [28]. The majority of these patients are managed with repeat dilations, depending on their complexity [29].

Reflux-induced esophageal stricture is generally treated via balloon dilation and continuous PPIs administration. However, most studies on reflux-induced esophageal strictures are case reports or reviews of case reports [24, 30]. In a prospective study, the authors reported no prophylactic effect of omeprazole (2 mg/kg) on post-esophageal atresia stricture, and no effect on reducing the number of endoscopic dilations [31]. For medication-refractory GERD patients, fundoplication may represent an important treatment option [7, 32]. LARS not only improved reflux, but also controlled reflux-related symptoms [33]. Thus, we believe that the treatment of esophageal strictures due to gastroesophageal reflux may require the surgical establishment of an adequate lumen and correction of the gastroesophageal reflux. Surgical treatment aims to dilate or resect the strictures, correct the gastroesophageal reflux, and repair the associated hernia, thus reversing the pathologic process. Hence, in the present study, we sought to examine the effects of dilatation therapy combined with PPIs and simultaneous therapy involving dilatation and LARS for esophageal stricture caused by GERD. According to the patient's individual wishes, the enrolled patients were assigned to two treatment groups: BD-PPIs group and BD-LF group.

Endoscopic dilatation was previously found to be successful in > 80–90% of cases, recurrent strictures occur within 1 year in one-third of cases [28]. Our results showed that the incidence of success was 57.7% in the BD-PPIs group and 80.4% in the BD-LF group in a 2-year follow-up. At 3 months and 24 months after surgery, a significant improvement in DMS, the number of refluxes with pH < 4, and LESP was observed in the BD-LF group (Table 2). However, in the BD-PPIs group, these parameters were not significantly changed compared with preoperative, and the incidence of reflux esophagitis was higher in the BD-PPIs group than in the BD-LF group. This showed that BD-LF had a better therapeutic effect than BD-PPIs. Moreover, the patients in the BD-PPIs group required more dilation sessions to achieve successful dilation as compared to those in the BD-LF group (*P* < 0.001, Table 3). Effective control of reflux mechanisms is the key factor for the successful management of GERD-related diseases [33]. LARS should be routinely performed in reflux-related benign esophageal strictures.

This study has several limitations. First, the sample size was small, so subgroup analysis was not possible. Second, we did not analyze other factors that might affect the results, such as alcohol, smoke, and eating habits. Finally, comparison of the outcomes of the surgical treatment of similar patient groups may be less valuable, if the evaluation is based on different scoring systems for the same problem.

In conclusion, dilatation of reflux-induced benign esophageal strictures was a common nonsurgical treatment, and anti-reflux surgery was recommended effective, and safe option. Future studies should optimize the therapy to establish the best means of preventing stricture formation in patients at risk of developing esophageal strictures. Further multi-center randomized controlled research is required to confirm these conclusions.
